# Preparation and Properties of Self-Healing Waterborne Polyurethane Based on Dynamic Disulfide Bond

**DOI:** 10.3390/polym13172936

**Published:** 2021-08-31

**Authors:** Gongbo Ye, Tao Jiang

**Affiliations:** Hubei Collaborative Innovation Center for Advanced Organic Chemical Materials, Ministry of Education Key Laboratory for the Green Preparation and Application of Functional Materials, School of Materials Science and Engineering, Hubei University, Wuhan 430062, China; 2015011113000048@stu.hubu.edu.cn

**Keywords:** waterborne polyurethane, self-healing, dynamic disulfide bond

## Abstract

A self-healing waterborne polyurethane (WPU) materials containing dynamic disulfide (SS) bond was prepared by introducing SS bond into polymer materials. The zeta potential revealed that all the synthesized WPU emulsions displayed excellent stability, and the particle size of them was about 100 nm. The characteristic peaks of N-H and S-S in urethane were verified by FTIR, and the chemical environment of all elements were confirmed by the XPS test. Furthermore, the tensile strength, self-healing process and self-healing efficiency of the materials were quantitatively evaluated by tensile measurements. The results showed that the self-healing efficiency could reach 96.14% when the sample was heat treated at 70 °C for 4 h. In addition, the material also showed a good reprocessing performance, and the tensile strength of the reprocessed film was 3.39 MPa.

## 1. Introduction

To date, polymer materials have been widely used in various fields because of their excellent corrosion resistance and mechanical and barrier properties. However, it is accessible to produce micro-slaps on the surface and inside of the traditional polymer materials due to the influence of mechanical, light and chemical substances in the manufactural processing and practical applications. These cracks are difficult to detect with the naked eye and may cause further damage to the materials, thus reducing the mechanical properties and safety of the materials and shortening their service time. Therefore, some researchers introduced the biological self-healing characteristics and mechanisms into polymer materials and formed functional self-healing materials through bionic design. These materials can recover their original properties spontaneously after damage or under external stimulation, resulting in new intelligent/smart materials with longer lives and more reliable performances, such as shaped memory polymer [1,2,3] and self-healing polymer [4,5] intelligent polymer materials.

From the perspective of bionics, self-healing materials can be divided into extrinsic and intrinsic types according to whether they contain additional repair reagents or not. Extrinsic self-healing can preinstall some special structural components (such as microcapsules or micro-vessels) in the polymer composites by implantation technology. When the material is damaged, the repair reagent at the damaged part is released rapidly, which promotes the polymerization reaction and repairs the material structure in time [6], whereas intrinsic self-healing materials can achieve multiple internal repairs through the breaking and recombination of reversible chemical bonds within or between molecules. Therefore, it is not necessary to add additional repair reagents in advance [7,8].

As the earliest discovered dynamic polymerization reaction, disulfide exchange reaction can be carried out at a low temperature and has great advantages in preparing dynamic polymers [9,10] For instance, aromatic disulfide compounds can form a rapid self-healing system [11]. In 2007, Nitschke et al. found that disulfide bonds in aromatic disulfides were easier to reach the translocation exchange equilibrium than that in aliphatic disulfides [12]. Ibon Odriozola et al. successfully prepared a catalyst-free disulfide bond polymer elastomer that can be repaired at room temperature by the condensation reaction of 4, 4′-dithiodianiline with isocyanate [13]. Kim et al. developed a transparent and easy-to-process polyurethane elastomer (IP-SS), which can be rapidly self-healed at room temperature and possess a maximum tensile strength of 6.8 MPa [14]. Zhang et al. prepared a self-healing polyurethane material based on solar light-induced disulfide bond metathesis reaction [15]. In addition to the electron-donating groups connected with disulfide bonds, it helps to reduce their bond energy and form hydrogen bonds between macromolecular chains, the amorphous structures of aliphatic monomers and soft and hard segments also play a key role in the self-healing of hydrogen sulfide. However, there are still some problems in the development of the disulfide exchange reaction. Aromatic disulfide monomers are usually expensive and not conducive to large-scale industrial production. At the same time, the molecular design usually leads to the yellowish appearance and low transparency of self-healing materials.

Herein, the low-cost aliphatic bis (2-alkylethyl) disulfide (HEDS) with flexible segments was used to prepare the self-healing waterborne polyurethane materials, which was synthesized by introducing HEDS-containing −OH groups into the WPU and reacted with −NCO groups. The successful introduction of SS bond in the polymer chain was verified by FTIR. Then, the influence of SS bond content on the self-healing efficiency of WPU film was studied by the tensile test, the dispersion and stability of WPU were assessed by the particle size and zeta potential analysis and the thermal properties of the polymer were tested by DMA and TGA. In addition, the reprocessing performance of the film was investigated by a plate vulcanizing machine.

## 2. Materials and Methods

### 2.1. Materials

Poly-tetrahydrofuran (PTMG, Mn = 1000), Triethylamine (TEA), 2,2-Dimthylolpropionic acid (DMPA), Dibutyltin dilaurate (DBTDL) and bis (2-alkylethyl) disulfide (HEDS) were obtained from Shanghai Aladdin Company, Shanghai, China. Hexamethylene diisocyanate (HDI) was purchased from Bayer Technology, Germany. PTMG and DMPA should be dehumidified under vacuum at 100 °C for 2 h before the experiment.

### 2.2. Preparation of WPU-SS Emulsion

WPU-SS was synthesized by three steps, in which acetone was replaced by DMAc to reduce the viscosity of polyurethane prepolymer. Table 1 lists the experimental formulation of different WPU samples. The molar ratio of the isocyanate group to hydroxyl group was kept at 1.4, and the ratio of PTMG to HEDS was 1/2 (WPU1), 1/1(WPU2) and 2/1(WPU3).

**Figure 1:**
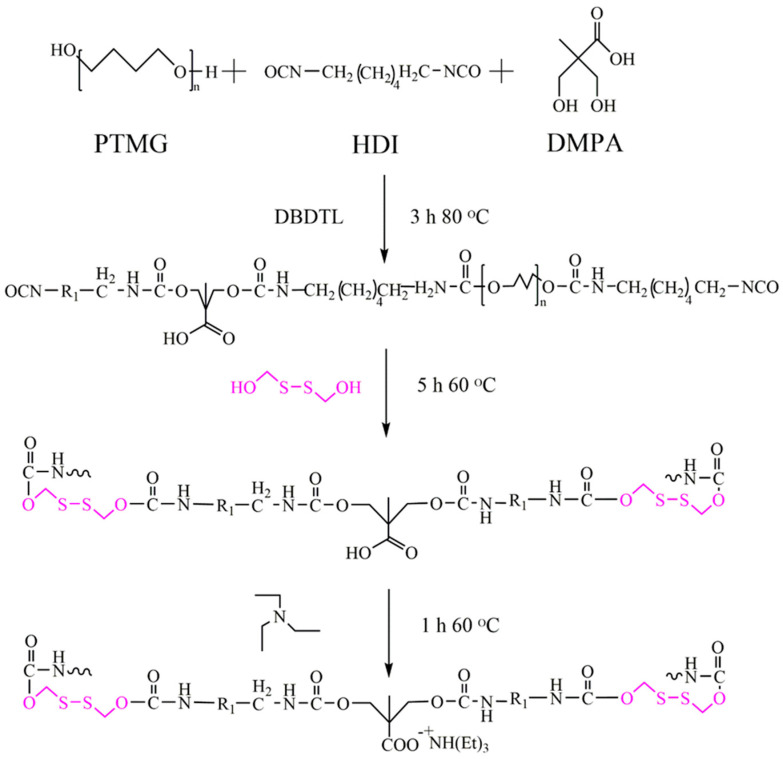
The synthesis process of WPU2 (R1 is the repeating chain segment).

According to the experimental formulation listed in Table 1, the synthesis process of WPU2 was illustrated. As shown in Figure 1, firstly, PTMG and DMPA were added in a 500-mL four-necked round-bottomed flask with a mechanical agitator, nitrogen inlet and drying condenser tube. DMAc was used as a solvent, and DBTDL was used as a catalyst. IPDI was slowly added after thoroughly stirring. Under mechanical stirring, the reaction temperature was 80 °C, when the content of −NCO reached the theoretical value, the polyurethane prepolymer terminated with −NCO was generated. Then, the temperature was reduced to 60 °C, HEDS containing −OH was added and reacted with isocyanate under nitrogen protection until the content of −NCO in the system reached the theoretical value again.

TEA with DMPA and other substances was added to neutralize −COOH in DMPA, and the system temperature was further reduced to 6~10 °C. The synthesized mixture was added to deionized water for high-speed dispersion for 1 h to obtain the final WPU2 emulsion. In the emulsification process, the residual −NCO of the prepolymer reacted with deionized water to form urea and biuret derivatives. The final WPU2 emulsion was light blue and the solid content was about 30 wt%. The WPU samples with PTMG and HEDS ratios of 1/2 and 2/1 were synthesized by the same method.

### 2.3. Preparation of WPU Films

The predetermined amount of WPU-SS emulsion was poured into the horizontally placed PTFE mold with the size of 10 cm × 10 cm × 1.5 cm. After drying at room temperature for 48 h, most of the water evaporated. Then, the film was stripped from the mold, and the water and solvent were completely removed within 24 h in a vacuum drying oven at 60 °C to obtain a constant weight WPU-SS film. The prepared WPU-SS films were stored in the dryer containing silica gel before further characterization.

### 2.4. Measurement of Isocyanate Content by Dibutyl-Amine Method

(1) Reagent preparation:
  - Preparation of 0.1 mol/L hydrochloric acid standard solution and calibration with anhydrous sodium carbonate.
  - Configure 0.1-mol/L di-n-butylamine-acetone solution: 12.9-g di-n-butylamine was placed in a 1000-mL volumetric flask, diluted with acetone and shaken.
  - Bromocresol green indicator: 0.1-g bromocresol green was dissolved in 100-mL volumetric flasks with a 1.5-mL concentration of 0.1-mol/L sodium hydroxide solution and diluted with distilled water to scale.
(2) Operation process: Accurate weighing 1~3-g prepolymer to 150-mL conical flask, adding 20-mL dibutyl-amine-acetone solution, fully reaction 20 min after adding 5 drops of bromocresol green indicator. Use the configured 0.1-mol/L hydrochloric acid standard solution to titrate the prepolymer. When the color changes from blue to yellow, it will be the end of the reaction. Read the volume and do a blank control experiment.
(3) Isocyanate content was determined by using the following Equation (1):

−NCO % = [(*V*_0_ − *V*) × c × 4.202]/m × 100% (1)

Thereinto, c is HCL concentration (mol/L), *V* is the sample consumed HCL volume (ml), *V*_0_ is blank consumed HCL volume (mL) and m is Sample mass (g).

### 2.5. Characterization

#### 2.5.1. Determination of Dispersion Stability

The average particle size and polydispersity index (PDI) of the dispersions were measured by dynamic light scattering (DLS) at room temperature using Zetasizer Nano ZS90 of Malvern Instrument and Equipment Company, Malvern, UK. In order to test the storage stability, all dispersions were stored in closed bottles and stored at room temperature.

#### 2.5.2. Fourier-Transform Infrared Spectroscopy (FTIR)

The synthesized polymer was analyzed by TENSORII Fourier-transform infrared spectrometer produced by BRUKER company in Germany. The attenuated total reflection (ATR) mode was used for infrared testing of WPU-SS samples. The spectra obtained were recorded in the range of 4000–500 cm^−1^.

#### 2.5.3. Dynamic Thermomechanical Analysis (DMA)

The dynamic mechanical properties of the rectangular WPU-SS spline (30 mm × 10 mm) were tested using the tensile mode of the DMA 850 dynamic thermomechanical analyzer of the United States TA company. The test temperature range is −100~120 °C, and the experimental frequency is 1 Hz. The heating rate is 5 °C/min.

#### 2.5.4. Thermogravimetric Analysis (TGA)

A certain amount of WPU-SS was investigated by the TGA55 thermogravimetric analyzer of TA company. It was carried out in a nitrogen atmosphere with an airflow velocity of 20 mL/min, a heating rate of 10 °C/min and a temperature range of 30~800 °C.

#### 2.5.5. X-ray Photoelectron Spectroscopy (XPS)

The surface chemical compositions of WPU-SS samples were analyzed by XPS ESCALAB 250A of Thermo Electron Corporation company with an Al Kα excitation radiation. The containment C 1s hydrocarbon peak at 284.8 eV was applied to calibrate the binding energies.

#### 2.5.6. Determination of Self-Healing Performance

The samples were cut into dumbbell-shaped samples, and five points were randomly selected on the dumbbell-shaped samples to measure the thickness using a desktop thickness gauge, and the average value was taken. The USA Instron 3369 universal tensile testing machine was used to test the tensile strength and elongation at break at 100 mm/min, and the average value of each sample was measured after 5 times.

The dumbbell-shaped standard sample was cut from the middle position to part of the adhesion, and then the incision part was docked together. The samples were repaired under different conditions. The tensile properties of original and repaired samples were tested, and the self-healing efficiency was calculated by using the following Equation (2):

η% = σ/σ_0_ × 100% (2)

where η is self-healing efficiency, σ is fracture strength of repaired specimen and σ_0_ is fracture strength of the original sample.

## 3. Results and Discussion

### 3.1. Dispersion and Stability Analysis

**Figure 2:**
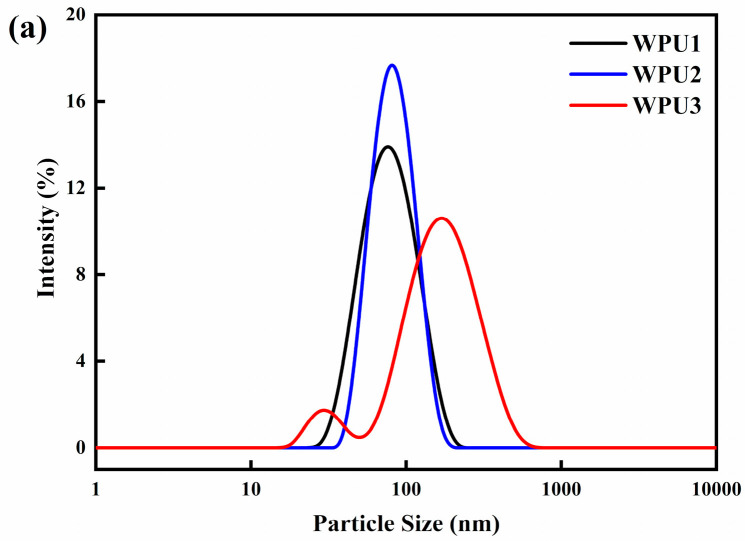

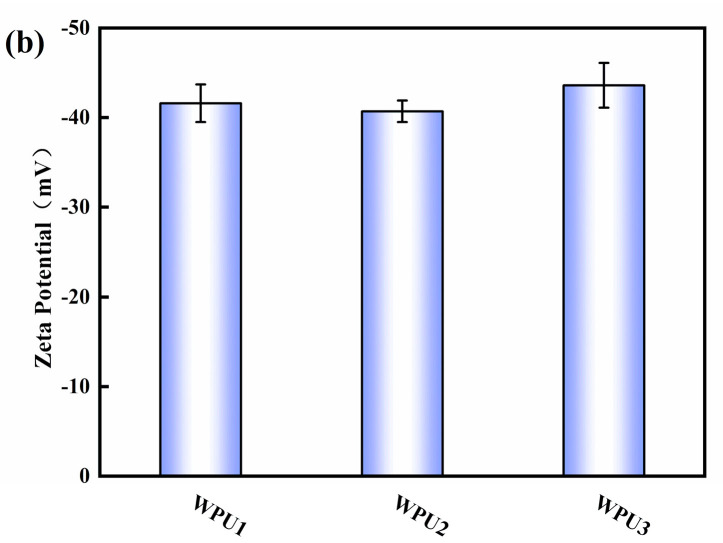
Particle size and distribution of WPU (**a**) and their zeta potential (**b**).

The synthesized WPU-SS emulsion was analyzed by a nano-particle size analyzer to evaluate its storage stability. The particle size and distribution of suspension waterborne polyurethane particles are shown in Figure 2a. The average particle sizes of WPU1, WPU2 and WPU3 were 71.27, 77.12 and 131.6 nm, respectively, and the PDI were 0.130, 0.084 and 0.285, respectively. The particle sizes of WPU1 and WPU2 dispersion systems are similar, and the polydispersity index shows that the distribution of WPU2 dispersion is more uniform than that of WPU1 dispersion. With the increase of soft segment content, the proportion of hydrophilic groups in WPU3 emulsion decreased, the polydispersity index increased, and the dispersion became worse.

Zeta potential is another important indicator for evaluating emulsion stability. As shown in Figure 2b, the Zeta potential of WPU1, WPU2 and WPU3 were −41.6, −40.7 and −43.6 mV, respectively. The high absolute value of zeta potential indicates that there is abundant electrostatic repulsion on the surface of water particles. Besides, introducing SS bonds in the WPU-SS chain had little effect on the Zeta potential value, manifesting that the addition of SS bonds had no significant effect on the stability of the emulsion. In addition, there is no deposit at the bottom of WPU-SS dispersions stored in closed glass bottles at room temperature for 2 months. The WPU emulsions were diluted into 1 wt% water dispersion and looked light blue. In summary, stable WPU emulsions can be obtained by introducing SS bond into the molecule chain.

### 3.2. Structure Analysis

**Figure 3:**
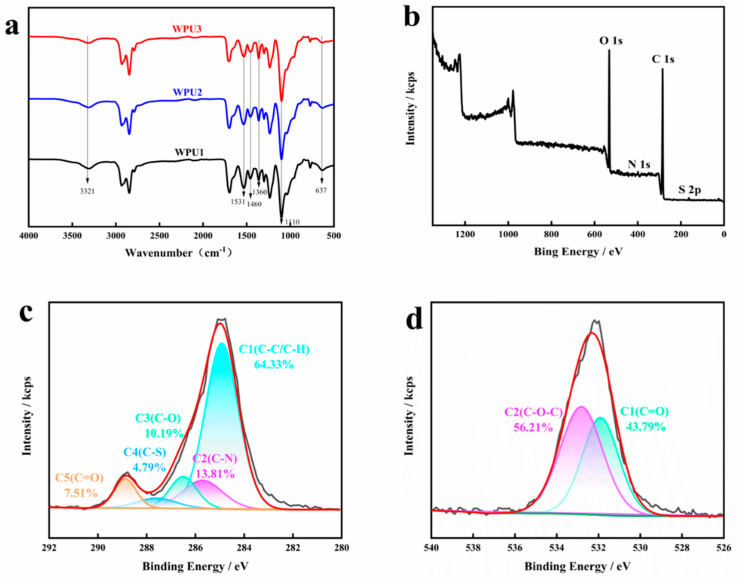
(**a**) FTIR spectra of WPU-SS samples, (**b**) XPS survey spectra of WPU2, (**c**) C 1s of WPU2, and (**d**) O 1s of WPU2.

Figure 3 are FTIR spectra and XPS survey spectra of the linear WPU-SS samples. In Figure 3a, all curves have no absorption peak at 2270 cm^−1^, which indicates that −NCO groups in the system are all involved in the reaction [16,17]. Compared with the three curves, the distribution of all characteristic peaks was essentially the same, indicating that −OH and −NCO on HEDS reacted to form carbamate, and no new chemical bond was formed. The three curves are in line with the characteristic peaks of waterborne polyurethane. The band located at 3321 cm^−1^ corresponds to the N−H stretching vibration and the band at 1531 cm^−1^ is assigned to the N–H in-plane bending vibration [18,19]. The absorption bands at 1460 and 1360 cm^−1^ are assigned to the –CH_2_– bending vibrations. The bands at 2940 and 2856 cm^−1^ are associated with the C–H asymmetry and symmetric stretching vibration of methylene in polyurethane molecular chain [20,21]. The band at 1110 cm^−1^ corresponds to bending vibration of C−O (aliphatic ether) in polyether polyol [22]. The characteristic band about 1707 and 1631 cm^−1^ is related with nonhydrogen bond C=O hydrogen bond C=O in urea, respectively [23,24,25]. HEDS in polymer chain has a characteristic absorption peak of SS bond at 637 [26]. With the increase of disulfide bond content, the absorption peak of the corresponding SS bond at 637 cm^−1^ became more obvious, which proved that SS bond was successfully introduced into waterborne polyurethane to form WPU-SS.

The XPS measurement was used to further confirm the construction by analyzing the chemical compositions in the WPU-SS system. The peaks in XPS survey spectrum indicated the present of S element (163.5 eV) in the samples due to disulfide bond should be successfully chemically linked to the matrix of waterborne polyurethane. In addition, the C 1s spectrum was further measured to explore the types of carbon bonds to analyze the existence of interfacial interactions, as shown in Figure 3c. The C 1s peak was curved-fitted into four main components including C-C/C-H, C-N, C-O, C-S and C=O bonds corresponding to the peaks at 284.8, 285.7, 286.5, 287.7 and 288.8 eV, respectively. The O 1s peak was curved-fitted into four main components including C-O-C and C=O bonds. The peak of C-S bond appeared implying that disulfide bond was successfully grafted onto polyurethane chains, as supported by FTIR results.

### 3.3. Dynamic Thermodynamic Analysis

**Figure 4:**
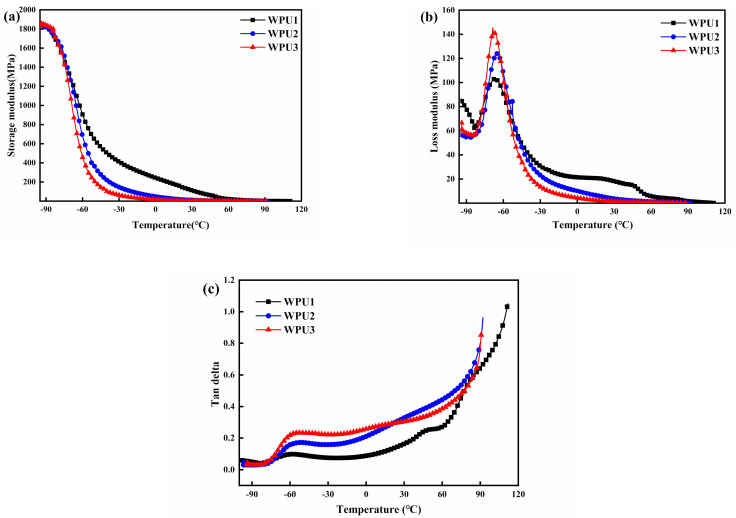
(**a**) Storage modulus, (**b**) loss modulus and (**c**) tan delta of WPU-SS samples with different contents of SS as a function of temperature.

The DMA results are shown in Figure 4. During the heating process of WPU-SS at −100~120 °C, the polymer with microphase separation structure showed two glass transition temperatures. The storage modulus and loss modulus of WPU-SS films are shown in Figure 4a,b, respectively. It could be found that the storage modulus (E′) decreased significantly at about −70 to −60 °C, while the loss modulus (E″) increased significantly at the same temperature range, which corresponding to the glass transition of the hard segments, moreover, with the increase of SS bonds, the E’ did not change much while the peak value of E″ changed obviously, which meant the move of the polymer chains took more energy. The decrease of disulfide bond content leads to the increase of soft segment content, which reduced the E′ and E″ of the films at room temperature. This is mainly due to the less restriction of the slip of the soft segment molecular chain, which leads to the easier entanglement of the molecular chain and the easier interaction with the main chain [27]. At the same time, the degree of microphase separation is reduced but the damping performance is improved.

The link between tan delta and temperature is determined in Figure 4c. With the loss of SS bond loading, the soft segment rises, and the microphase separation diminishes gradually. When the microphase separation is cut down to a certain extent, the glass transition temperature of the hard segment cannot be shown in the experiment. Since the glass transition temperature of the hard segment is much lower than room temperature, the film is in a highly elastic state and exhibits the properties of an elastomer at room temperature.

### 3.4. Thermal Stability Analysis

**Figure 5:**
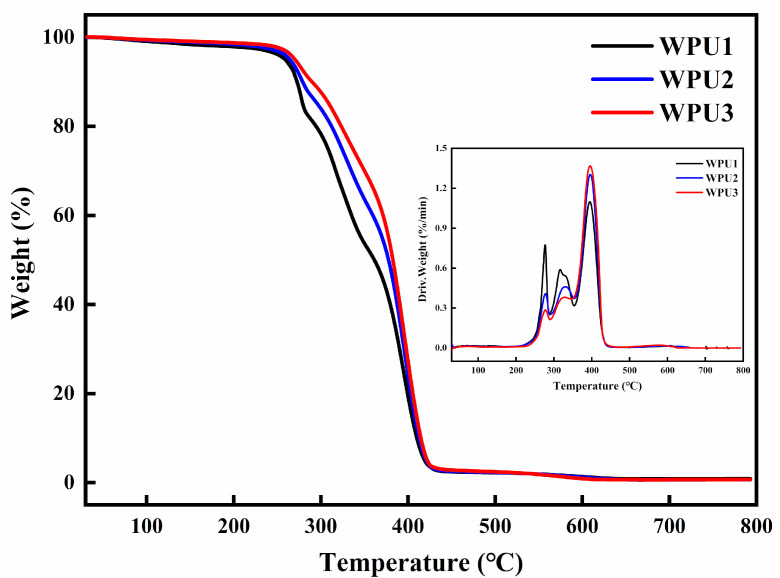
TGA curves and DTG curves of different WPU-SS films.

To investigate the thermal stability of WPU-SS films, thermogravimetric analysis (TGA) was carried out at 30~800 °C in a nitrogen atmosphere. Figure 5 shows the typical TGA and the first derivative curve DTG, respectively. The characteristic thermal degradation data display in Table 2. From the TGA curve, the thermal stability of WPU-SS films was improved with the decrease of short chain disulfide bond content and the enhancement of long chain PTMG content. In particular, the 50% weight loss temperature (T_d_50%) increased from 361 °C to 382 °C. In the region of 230~440 °C, there are two obvious degradation stages for WPU-SS films. The partial weight loss at 230~350 °C can be attributed to the rupture of the amino ester bond in the hard segment isocyanate, and the thermal stability of the hard segment is poor, which can be decomposed into primary amines (or secondary amines), alkenes and carbon dioxide [28,29,30]. In contrast, polyether has better thermal stability, 350~440 °C weight loss is mainly the decomposition of the soft segment [31,32,33].

### 3.5. Tensile Properties and Self-Healing Efficiency of WPU-SS Films

**Figure 6:**
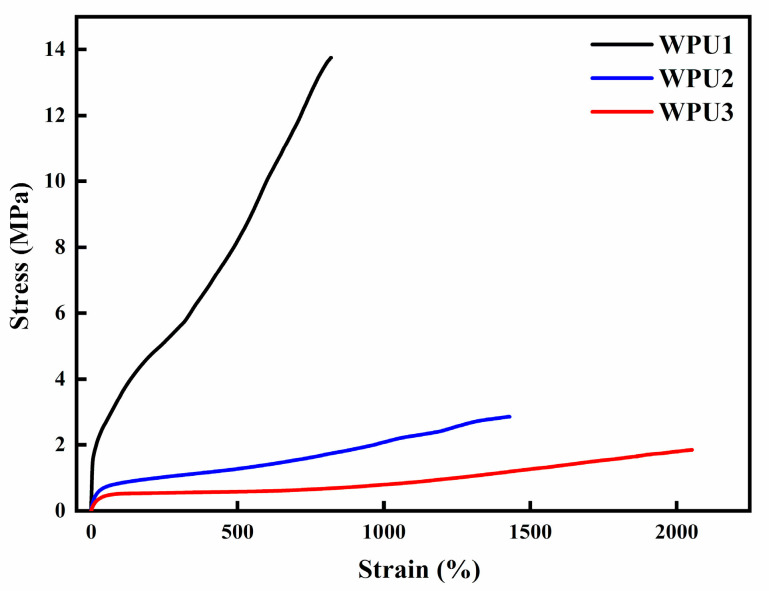
Stress-strain curve of WPU1, WPU2, WPU3.

In addition, the self-healing process of WPU films was evaluated more quantitatively through tensile measurement, and the self-healing efficiency was calculated according to the final tensile stress ratio of the repaired sample to the original sample. Figure 6 reveals the stress-strain curves of the original samples of WPU1, WPU2 and WPU3. The film of WPU1 exhibits a high tensile strength since the hard segment content is relatively high, and the content of carbamate in WPU1 may be more than that in WPU2 and WPU3. On the contrary, WPU2 and WPU3 possesses high soft segment content, and strong molecular weight mobility, so its self-healing performance is also relatively improved.

**Figure 7:**
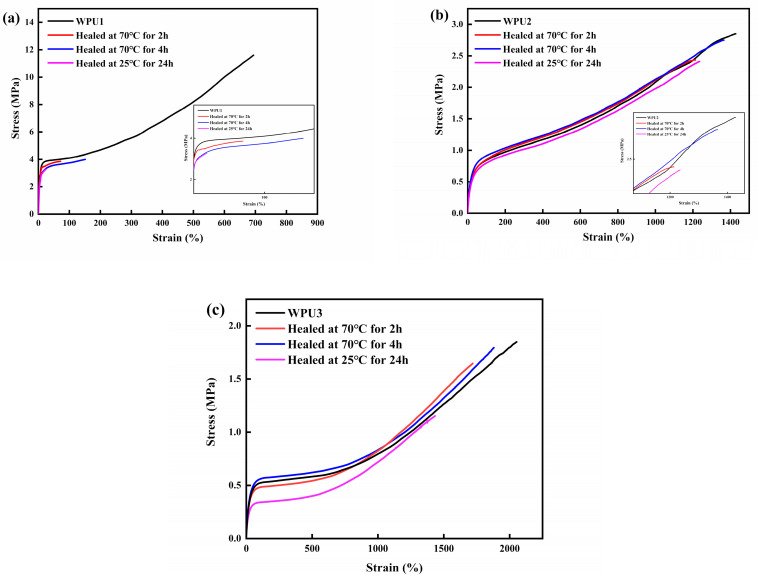
Stress-strain curves of WPU1 (**a**), WPU2 (**b**) and WPU3 (**c**) films repaired under different conditions.

Figure 7 shows the self-healing efficiency of WPU1 WPU2 and WPU3 under different conditions. The self-healing efficiency of WPU1 is only 29.02% after 4 h at 70 °C, while the healing efficiency of the WPU2 and WPU3 films reached 96.14% and 97.28% under the same conditions, respectively. Meanwhile, WPU2 and WPU3 films also had a self-healing efficiency of up to 84.21% and 85.86% after being placed at 25 °C for 24 h. The table of repair efficiency changing over time is shown in Table 3. There is no doubt that introducing SS bond endowed the film with excellent self-healing performance. Of course, the content of the soft segment also exerts a certain degree of influence on the repair effect of the film [34,35].

### 3.6. Reprocessing Performance

**Figure 8:**
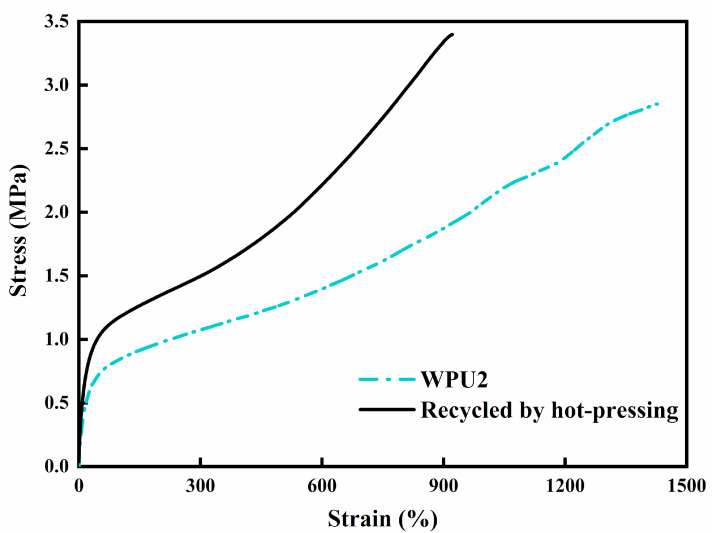
Tensile properties of the reprocessed WPU2 film.

After being placed in a constant temperature and humidity box for 24 h, the cut dumbbell-shaped spline was used for the tensile test. Figure 8 provides the stress-strain curve of WPU2 film and WPU2 film after reprocessing. The tensile strength of WPU2 film after reprocessing was 3.39 MPa, compared with the original spline, its tensile strength increased and the elongation at break reduced, which indicated that the film had a good reprocessing performance.

## 4. Conclusions

In this paper, based on the principle of introducing reversible covalent bond containing SS into polymer, functional self-healing waterborne polyurethane was synthesized with PTMG as soft segment, IPDI as hard segment and DMPA containing hydrophilic group as auxiliary. The zeta potential revealed that all the synthesized WPU emulsions displayed excellent stability, and the particle sizes of WPU1 and WPU2 emulsions were relatively small. The characteristic peaks of N-H and S-S in urethane were verified by FTIR. Furthermore, the tensile strength, self-healing process and repair effect of WPU-SS film were quantitatively evaluated by tensile measurement. After the cut spline WPU2 sample was heat treated at 70 °C for 4 h, it could maintain the tensile strength and the self-healing efficiency could reach 96.14%, whilst the repair efficiency of the sample was 84.21% after 24-h self-repair at 25 °C. In addition, to realize the reprocessing of the self-healing film, the cut WPU2 film fragments were hot pressed at 130 °C for 20 min to reshape into a square film by a flat vulcanizing machine. The results indicated that the tensile strength of the reprocessed WPU2 film was 3.39 MPa, and it possessed good reprocessing performance.

## Figures and Tables

**Table 1:** Molar ratio formulations of different WPU samples.

	HDI	PTMG	DMPA	HEDS	TEA
WPU1	3.5	0.55	0.85	1.10	0.85
WPU2	3.5	0.83	0.85	0.83	0.85
WPU3	3.5	1.10	0.85	0.55	0.85

**Table 2:** Particular thermal degradation dates of diifferent WPU samples.

	T_d_5% (°C)	T_d_10% (°C)	T_d_50% (°C)
WPU1	256	273	361
WPU2	264	278	377
WPU3	270	290	382

**Table 3:** Details of Tensile Results and the Healing Efficiency of WPU-SS Samples under different conditions.

Samples	Heat Treatment Time (h)	Tensile Strength (MPa)	Elongation at Break (%)	Healing Efficiency (%)
WPU1	/	13.75	820.01	/
	2	3.85	71.67	28.00
	4	3.99	151.12	29.02
	24(25 °C)	3.26	23.9	23.70
WPU2	/	2.85	1427.78	/
	2	2.43	1213.34	85.26
	4	2.74	1365.01	96.14
	24(25 °C)	2.40	1235.01	84.21
WPU3	/	1.84	2053.08	/
	2	1.64	1719.64	89.13
	4	1.79	1880.01	97.28
	24(25 °C)	1.58	1671.88	85.86

## Data Availability

Not applicable.
